# Analysis of cardiac signals using spatial filling index and time-frequency domain

**DOI:** 10.1186/1475-925X-3-30

**Published:** 2004-09-10

**Authors:** Oliver Faust, Rajendra Acharya U, SM Krishnan, Lim Choo Min

**Affiliations:** 1 Dept. of ECE, Ngee Ann Polytechnic, Singapore 599489; 2 Dept. of Biomedical Engineering, Nanyang Technological University, Singapore

## Abstract

**Background:**

Analysis of heart rate variation (HRV) has become a popular noninvasive tool for assessing the activities of the autonomic nervous system (ANS). HRV analysis is based on the concept that fast fluctuations may specifically reflect changes of sympathetic and vagal activity. It shows that the structure generating the signal is not simply linear, but also involves nonlinear contributions. These signals are essentially non-stationary; may contain indicators of current disease, or even warnings about impending diseases. The indicators may be present at all times or may occur at random in the time scale. However, to study and pinpoint abnormalities in voluminous data collected over several hours is strenuous and time consuming.

**Methods:**

This paper presents the spatial filling index and time-frequency analysis of heart rate variability signal for disease identification. Renyi's entropy is evaluated for the signal in the Wigner-Ville and Continuous Wavelet Transformation (CWT) domain.

**Results:**

This Renyi's entropy gives lower 'p' value for scalogram than Wigner-Ville distribution and also, the contours of scalogram visually show the features of the diseases. And in the time-frequency analysis, the Renyi's entropy gives better result for scalogram than the Wigner-Ville distribution.

**Conclusion:**

Spatial filling index and Renyi's entropy has distinct regions for various diseases with an accuracy of more than 95%.

## Background

Bio-signals are essentially non-stationary signals; they display a fractal like self-similarity. They may contain indicators of current disease, or even warnings about impending diseases. The indicators may be present at all times or may occur at random – in the time scale. However, to (study and) pinpoint anomalies in voluminous data collected over several hours is strenuous and time consuming. Therefore, computer based analytical tools for in-depth study and classification of data over day long intervals can be very useful in diagnostics.

Electrocardiography deals with the electrical activity of the heart. Monitored by placing sensors at defined positions on chest and limb extremities of the subject, electrocardiogram (ECG) is a record of the origin and propagation of the electric action potential through cardiac muscle. It is considered a representative signal of cardiac physiology, useful in diagnosing cardiac disorders. The state of cardiac health is generally reflected in the shape of ECG waveform and heart rate. It may contain important pointers to the nature of diseases afflicting the heart. However, bio-signals being non-stationary signals, this reflection may occur at random in the time scale.

(That is, the disease symptoms may not show up all the time, but would manifest at certain irregular intervals during the day.) Therefore, for effective diagnostics, the study of ECG pattern and heart rate variability signal (instantaneous heart rate against time axis) may have to be carried out over several hours. HRV is a useful signal for understanding the status of the autonomic nervous system (ANS).

The interest in the analysis of heart rate variability (HRV), that is, the fluctuations of the heart beating in time, is not new. And much progress was achieved in this field with the advent of cheap and massive computational power, which provoked many recent advances.

HRV is a non-invasive measurement of cardiovascular autonomic regulation. Specifically, HRV is a measurement of the interaction between sympathetic and parasympathetic activity in autonomic functioning. There are two main approaches for analysis: time domain analysis of HRV [for standard deviation of normal to normal intervals (SDNN)]; and frequency domain analysis [for power spectrum density (PSD)]. The latter provides high frequency (parasympathetic activity) and low frequency (sympathetic and parasympathetic activity) and total power (sympathetic/parasympathetic balance) values [1-3]. Recent results on HRV signal analysis show that its dynamic behavior involves non-linear components that also contribute in the signal generation and control [4]. The autonomic nervous system (ANS) modulates the cardiac pacemaker and provides beat-to-beat regulation of the cardiovascular rhythm. Application of wavelet transformation techniques to beat-to-beat heart rate variations (HRV) provides an important non-invasive tool for monitoring the autonomic nervous system functioning.

The cardiovascular system is a complex system that includes heart and vessels. ECG and HRV are two methods for study it. Hence, many attempts have been made to analyze these signals and extract information about the cardiovascular system. Most of the methods used are linear and it has been recognized that nonlinear methods may be more suitable for analyzing signals that originate from complex nonlinear living systems [5]. Recent developments in non-linear analysis have provided various methods for the study of the complex cardiovascular system [6]. It is now generally recognized that many processes generated by the biological system can be described in an effective way by using the methods of nonlinear dynamics. The nonlinear dynamical techniques are based on the concept of chaos, which was first introduced with applications to complicated dynamical systems in meteorology [7]. Since then, it has been applied to medicine and biology [8,9]. A particularly active area for the application of chaos theory has been cardiology [10,11], where many aspects have been addressed including whether chaos can be used to represent healthy or diseased state [12].

A complex system like cardiovascular system can not be linear in nature and by considering it as a nonlinear system can lead to better understanding of the system dynamics. Recent studies have also stressed the importance of nonlinear techniques to study HRV in both health and disease. The progress made in the field using measures of chaos has attracted scientific community applying these tools in studying physiological systems, and HRV is no exception. There have been several methods of estimating invariants from nonlinear dynamical systems reported in the literature. Recently, Fell et al and Radhakrishna et al have tried the nonlinear analysis of ECG and HRV signals respectively [13,14]. Also, Addison at al showed that coordinated mechanical activity in the heart during ventricular fibrillation may be made visible in the surface ECG using wavelet transform [15]. Rajendra et al, [16] have classified the HRV signals using Artificial Neural Networks (ANN) and Fuzzy equivalence relation. Recently, Renyi's entropy is used for texture analysis by Grigorescu et al [17]. Gokcay et al have applied Renyi's entropy to clustering and analyze the resulting staircase nature of the performance function that can be expected during learning [18]. In this work, different heart rate signals are analyzed using spatial filling index and time frequency techniques. Renyi's entropy is evaluated for the different cardiac abnormalities.

## Methods

ECG data for the analysis was obtained from MIT-BIH arrhythmia database [19]. Prior to recording, the ECG signals were processed to remove noise due to power line interference, respiration, muscle tremors, spikes etc. The R peaks of ECG were detected using Tompkins's algorithm [20]. The ECG data contains eight different classes representing eight different diseases. The number of datasets chosen for each of the eight classes is given in Table 1. The Normal class contains datasets from people where no cardiac abnormality was diagnosed. The remaining classes are named according to the diagnosed cardiac abnormality, *premature ventricular contraction *(PVC), *Complete Heart Block *(CHB), *Sick Sinus Syndrome *(SSS), *Congestive heart failure *(CHF), *Ishemic/Dilated cardiomyoapathy *(ISCDIL), *Atrial Fibrillation *(AF), and ventricular fibrillation (VF).

**Table 1:** Number of subjects in various groups

Type	Normal	PVC	CHB	SSS	CHF	ISCDIL	AF	VF
Number of datasets	60	60	20	20	40	20	35	45

Each dataset is taken consists of more than10,000 samples and the sampling frequency of the data is 360 Hz. The interval between two successive QRS complexes is defined as the RR interval (t_r-r _seconds) and the heart rate (beats per minute) is given as:

HR = 60/t_r-r _    (1)

### Spatial Filling Index

Let the signal be represented by the coordinates of a point *X*(*k*) in phase space. Then the dynamical behavior of the signal is reconstructed by succession of these points *X*(*k*) in the phase space. Phase space reconstructions are based on the analysis of dynamic systems by delay maps. The vectors *X*(*k*) in the multidimensional phase space are constructed by time delayed values of the time series, which determine the coordinates of the phase space plot.

*X*(*k*) = {*x *(*k*), *x*((*k *+ *τ*), ...,*x *(*k *+ (*E*-1)*τ*)} for *k *= 1,2,...,*N *- (*E *- 1)*τ *    (1)

where *X*(*k*) is one point of the trajectory in the phase space at time *k*, *x*(*k *+ *τ*) are the coordinates in the phase space corresponding to the time delayed values of the time series, *τ *is the time delay between the points of the time series considered and *E *is the embedding dimension, which is the number of coordinates of the phase space plot. The attributes of the reconstructed phase space plot depend on the choice of value of *τ*. One way to choose *τ *is to take it as the time it takes the autocorrelation function of the data to decay to 1/*e *[21]. Another method is to take the first minimum in the graph of average mutual information [22], which appears to be better since it considers the nonlinear structure in the signal. It has been established using this method that the value of 7 for *τ *is the best choice for ECG signals and 5 for HRV signals [23].

From the given signal *x *(1), *x*(2), ..., *x *(*N*), a matrix *A*_*E *_is obtained as



where *E *is the number of dimensions and *M *is related to *N *by the equation:

*M *= *N *- (*E *-1)*τ *    (3)

By plotting column 2 of matrix *A *against column 1 (for the case *E *= 2), the phase space plot for two dimensions is obtained.



Similarly, the first three columns of matrix *A*_3 _represent a phase space plot in three dimensions. Now, a normalized matrix *B*_*E *_is obtained by dividing each element of *A*_*E *_by *x*_max _where

*x*_max _= max |*x*(*k*)| 1 ≤ *k *≤ *N *    (5)

The matrix *B*_2 _(in two dimensions) is hence represented as



In two dimensions, the phase space plot corresponding to the normalized matrix spans from -1 to +1 on either axis. The phase space area is now divided into small square areas of size {*R × R *|*R *∈ *Real*, 2/R ∈ *Integer*}. Then the number of grids in the normalized phase space is *n *= 2/*R*. A matrix *C *is now obtained with its elements *c*(*i,j*) equal to the number of phase space points falling in a grid *g*(*i*,*j*). The matrix *C *is called the phase space matrix and its elements are divided by *m*, where



This division yields *P*(*i*,*j*), the probability of a phase space point falling in a grid *g*(*i*,*j*). A matrix *Q *is now formed by squaring each element of *P *to get *q*(*i*,*j*) as the elements of *Q*. The sum of elements of matrix *Q *is calculated as



The spatial filling index *η *is defined as:

*η *= *s */ *n*^2 ^    (9)

Now the value of *η *is used to quantify the degree of variability in the test signals.

### Time-Frequency analysis

There are three common approaches to generating the time-frequency (TF) plots. These are the short Time Fourier Transform; the Wigner-Ville based bilinear distributions and the Continuous Wavelet Transform. In this investigation the latter two were used.

### Wigner-Ville analysis

The Wigner-Ville distribution (WVD) is defined as:



where *z*(*t*) is the analytic signal and *h*(*τ*) is a window function. The results where obtained using a Hamming window. This window attenuates the interferences oscillating perpendicularly to the frequency axis. The WVD satisfies a large number of desirable mathematical properties. In particular, the WVD is always real-valued; it preserves time and frequency shifts and satisfies the marginal properties. Moreover, the WVD conserves the Energy of the signal. We obtain the Energy (*E*_*x*_) by integrating the WVD of *z *all over the time frequency plane:



With the Energy conservation property the WVD can be interpreted in terms of probability density: expression (10) is the Fourier transform of an acceptable form of characteristic function for the distribution of energy. Therefore, the WVD can be used to obtain the information content of a signal; this thought is further extended in Section 4.3.

### Continuous Time Wavelet Transform (CWT) analysis

A *'wavelet' *implies a small wave of finite duration and finite energy, which is correlated with the signal to obtain the wavelet coefficients [24]. The reference wavelet is known as the *mother wavelet*, and the coefficients are evaluated for the entire range of dilation and translation factors [25]. Initially the mother *wavelet *is shifted (translated) continually along the time scale for evaluating the set of coefficients at all instants of time. In the next phase, the wavelet is dilated for a different width – also normalized to contain the same amount of energy as the mother wavelet – and the process is repeated for the entire signal. The wavelet coefficients are real numbers usually shown by the intensity of a chosen color, against a two dimensional plane with y-axis representing the dilation (scaling factor) of the wavelet, and the x-axis, its translation (shift along the time axis). Thus the wavelet transform plot (*scalogram*) can be seen as a color pattern against a two dimensional plane. In the CWT the wavelet coefficients are evaluated for infinitesimally small shifts of translation as well as scale factors. That is, the color intensity of each pixel in the *scalogram *is separately evaluated, and the resulting pattern contains information about the size and location of the 'event' occurring in the time domain [26,27]. Since the dilated wavelet is normalized to contain the same amount of energy as the mother wavelet; the *scalogram *representation of even high frequency, low energy 'events' occurring in the time scale are more conspicuous than in the Fourier Transform. Thus the color patterns in the scalogram can be useful in highlighting the abnormalities specific to different types of disease. MATLAB version 6.1 is used to plot the various scalogram plots.

For a given wavelet *Ψ*_*a*,*b*_(*t*), the coefficients are evaluated using Eq. (12):



The wavelet, defined as ,...small wave of finite duration and finite energy...' has also zero mean value,  is energy normalizing coefficient, and *Ψ*_*a*,*b*_(*t*) is the mother wavelet; a → scale factor ; b → translation factor.

Just like the WVD, the CWT representation preserves also the energy of the signal. The total energy (*E*_*x*_) is obtained by integrating over all scale and translation factors:



The *scalogram *patterns thus obtained also depend on the wavelet chosen for analysis. Bio-signals usually exhibit self similarity patterns in their distribution, and a wavelet which is akin to its fractal shape would yield the best results in terms of clarity and distinction of patterns. In the present work, the analysis is based on the Morlet wavelet. This wavelet gives good result compared to all the other wavelets.

The Morlet wavelet function is given by:



### Renyi's Entropy (RE)

The previous sections detailed WVD and CWT as two methods to represent a signal in the time-frequency domain. This section is concerned with the interpretation of the time-frequency representation. The signals represent measurements taken from patients being either normal or suffering from different vascular diseases. The goal is to find a measure which allows classifying the different signals according to the medical conditions.

One interesting information that one may obtain from the time-frequency representation is the number of elementary signals present in the current observation. This leads to the following question: How much separation between two elementary signals must one achieve in order to be able to conclude that there are two signals present rather then one?

A solution to this problem is given by applying an information measure to a time-frequency distribution of a signal. This can be done, because CWT and WVD preserve the energy of the signal.

Unfortunately, the well known Shannon information can not be applied to the time-frequency representation of a signal, because it contains negative values. One information measure, which allows negative values in the distribution, is *Renyi's entropy*. This information measure was used to analyze the time-frequency representation of the measurement data.

Renyi's entropy definition is derived from his proposed theory of means [28]



where *φ*(.)- is a continuous and strictly monotonic function subclass of Kolmogorov-Nagumo functions. To satisfy the constraints of an information measure



*I*(*p*_*k*_)- any information measure

Simplifying the above relation, we have



The third order Renyi's entropy (*α *= 3) is calculated from the WVD time-frequency representations as follows:



similarly, the third order Renyi's entropy is calculated form the CWT as follows:



The result produced by this measure ( and ) is expressed in *bits*: If one elementary signal yields zero bit of information (2^0^), then two well separated elementary singles will yield one bit of information (2^1^), four well speared elementary singles will yield two bits of information (2^2^), and so on. It shows that for different cardiac signals the Renyi's entropy in the time-frequency domain is different.

### One-Way Analysis of Variance (ANOVA)

The purpose of one-way ANOVA is to find out whether data from several groups have a common mean. That is, to determine whether the groups are actually different in the measured characteristic.

One-way ANOVA is a simple special case of the linear model. The one-way ANOVA form of the model is where:

*y*_*ij *_= *α*_.*j *_+ *ε*_*ij*_

• *y*_*ij *_is a matrix of observations in which each column represents a different group.

• *α*_.*j *_is a matrix whose columns are the group means. (The "dot j" notation means that applies to all rows of the jth column. That is, the value *α*_*ij *_is the same for all i.)

• *ε*_*ij *_is a matrix of random disturbances.

The model posits that the columns of y are a constant plus a random disturbance. You want to know if the constants are all the same.

## Results

The result section compares the three different analyzing methods. Each of these methods results in a single parameter for each of the datasets. For Phase Space the parameter used for comparison is the spatial filling index (*η*) defined in Equation (9). For WVD the parameter is the third order Renyi's entropy () defined in Equation (14). For CWT the parameter is also the third order Renyi's entropy () defined in Equation (15). For each dataset these three parameters (,  and *η*) where calculated. Table 2 shows the mean and the variance (normalized by *N*-1 where *N *is the sequence length) of these parameters for each of the data classes. The p-value, also shown in Table 2, results form the ANOVA test for each of the parameters.

**Table 2:** Results for various cardiac abnormalities

Type	SSS	PVC	CHB	NORMAL	CHF	AF	ISCDIL	VF	p-value
*η *Phase Space	1.56 ± 0.08	3.77 ± 11.82	7.07 ± 0.57	6.76 ± 1.61	7.71 ± 0.20	2.26 ± 0.05	7.44 ± 1.24	3.77 ± 8.78	0.00005
Wigner-Wille	3.38 ± 2.74	4.79 ± 1.46	5.65 ± 0.61	4.00 ± 1.52	2.10 ± 0.61	3.31 ± 2.45	4.07 ± 2.78	4.44 ± 0.96	0.021
Scalogram	2.84 ± 1.89	2.25 ± 1.06	3.01 ± 0.29	1.67 ± 0.84	1.78 ± 0.82	2.04 ± 1.37	2.15 ± 0.57	3.29 ± 0.33	0.001

The proposed technique was applied to a number of different signals, both normal and abnormal. Some of the normal and abnormal signals used in the analysis, along with their two dimensional plots are shown in the Figures 1,2,3,4,5,6,7,8.

**Figure 1:**
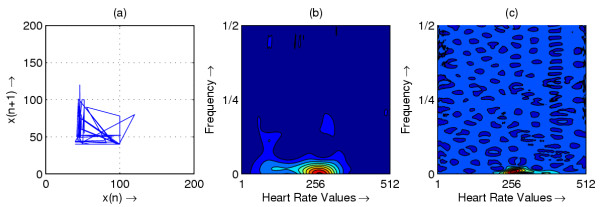
Heart rate in representative subject with SSS; (a) Phase space plot (b) Scalogram (c) Wigner-Ville distribution

**Figure 2:**
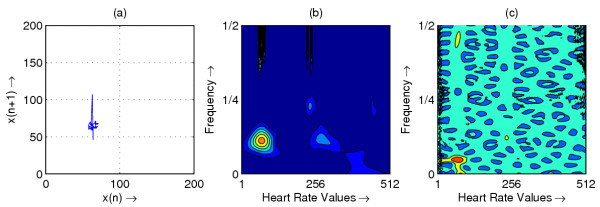
Heart rate in representative subject with PVC; (a) Phase space plot (b) Scalogram (c) Wigner-Ville distribution

**Figure 3:**
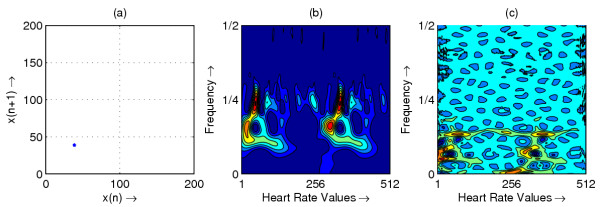
Heart rate in representative subject with CHB; (a) Phase space plot (b) Scalogram (c) Wigner-Ville distribution

**Figure 4:**
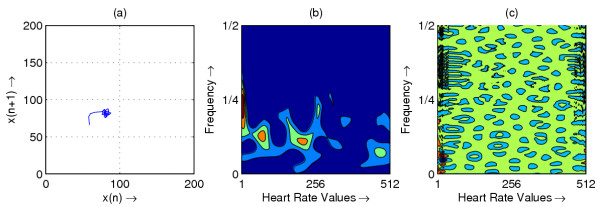
Heart rate in representative subject with Normal; (a) Phase space plot (b) Scalogram (c) Wigner-Ville distribution

**Figure 5:**
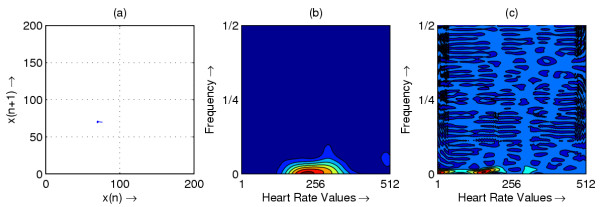
Heart rate in representative subject with CHF; (a) Phase space plot (b) Scalogram (c) Wigner-Ville distribution

**Figure 6:**
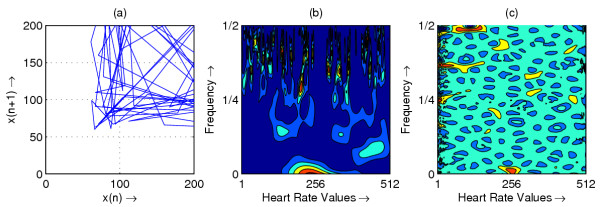
Heart rate in representative subject with AF; (a) Phase space plot (b) Scalogram (c) Wigner-Ville distribution

**Figure 7:**
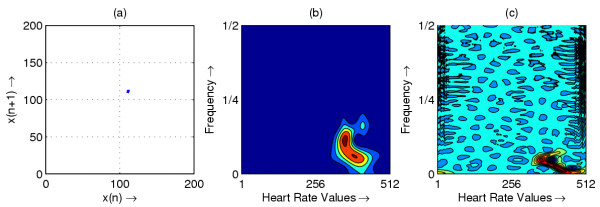
Heart rate in representative subject with Ishemic/Dilated Cardiomyopathy; (a) Phase space plot (b) Scalogram (c) Wigner-Ville distribution

**Figure 8:**
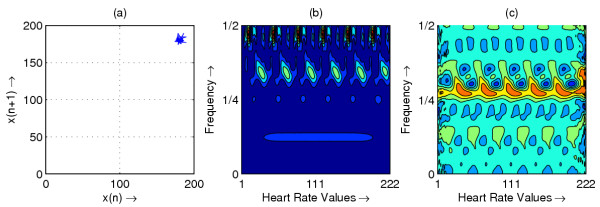
Heart rate in representative subject with VF; (a) Phase space plot (b) Scalogram (c) Wigner-Ville distribution

For the time frequency plots the normalized frequency is shown over the hart rate values. It is not useful to state an absolute frequency, because such a value is not relevant for the cardiac system under observation. Moreover, the relative frequency representation allows comparing the time frequency analysis results form varying observation intervals. As example, the observation interval for the VF data is significantly shorter as for the rest of the data, but still the results can be compared.

## Discussion

The resulting phase space plots for various types of disease are shown in Figure 1(a),2(a),3(a),4(a),5(a),6(a),7(a),8(a). In SSS – III (Sick Sinus Syndrome – III, Bradycardia-Tachycardia) there is a continuous variation of heart rate between Bradycardia and Tachycardia. The phase space plot spreads over a larger area (Figure 1(a)). In the Ectopic beat abnormality; there would be a sudden impulsive jump in the heart rate. This may be due to a Premature-Ventricular beat in the ECG signal. This is indicated as a sudden spike in the phase space plot (Figure 2(a)). In Complete Heart Block (CHB) cases, as the atrio-ventricular node fails to send electrical signals rhythmically to the ventricles, the heart rate remains low. The phase space plot reduces almost to a point, indicating very little change with time (Figure 3(a)). For Normal cases, the phase space plot looks like a cluster of points (Figure 4(a)). In the Congestive heart failure (CHF), the heart rate variation is lower and hence the phase space plot spread in a very small area (Figure 5(a)). In the Atrial Fibrillation (AF), heart rate signal records highly erratic variability; this is depicted as scattering of points in the phase space plot (Figure 6(a)). In the case of Ischemic/Dilated cardiomyopathy, the ventricles are unable to pump out blood to the normal degree. Here the heart rate variation is very small. And hence the phase space plot will be almost a point (Figure 7(a)). And its phase space plot resembles that of Normal class. Finally, in VF, the heart rate variation is high and hence the phase space plot is randomly distributed (Figure 8(a)).

The contour plots of scalogram and Wigner-Ville distribution plot for the different abnormalities are shown in figures 1(b),1(c),2(b),2(c),3(b),3(c),4(b),4(c),5(b),5(c),6(b),6(c),7(b),7(c),8(b),8(c) respectively. In the contour plot of scalogram (Figure 1(b)), for SSS, there is clear indication of variation of high frequency and low frequency in the form of irregular circles at these frequencies. In PVC (Figure 2(b)), a irregular circle is shown at high frequency indicating the spike of the signal. These irregular circles or contours are at low frequencies for CHB (Figure 3(b)), indicating smaller R-R variation. In normal case (Figure 4(b)) these contours are in the middle frequency due to variation in the R-R interval. In CHF (Figure 5(b)) and Ischemic/Dialted cardiomyopathy (Figure 7(b)), the R-R variation is extremely low. Hence the contours are aligned at the low frequency. In AF (Figure 6(b)), due to very high R-R variation are shown as irregular contours at high frequency. For VF, this R-R variation is slightly low and as result the contours are aligned at the middle of the contour plot (Figure 8(b)).

The contour plots of the Wigner-Ville distribution does not indicate as clearly as contour plot of scalogram for various cardiac diseases.

The spatial filling index decreases or increases from the normal class for the abnormal subjects in different ranges (Table 2) depending on the R-R variation. This value decreases for the abnormalities of high R-R variation and increases for CHB, CHF and Ishemic/Dilated cardiomyoapathy, which has low R-R variation. This parameter has excellent 'p' value for various classes (0.00005). The Renyi's entropy has high value for cardiac abnormalities like Ischemic/Dilated cardiomyopathy, CHB, VF and it decreases for Normal, PVC, AF, SSS and CHF. This RE gives lower 'p' value for scalogram than Wigner-Ville distribution and also, the contours of scalogram visually show the features of the diseases. Hence, in the time-frequency analysis, the Renyi's entropy gives better result for scalogram than the Wigner-Ville distribution.

## Conclusion

Considering heart as a nonlinear complex system and processing various cardiovascular signals (HRV) seems to provide very useful information for detection of abnormalities in the condition of the heart that is not available by conventional means. In this paper, a phase space and the time-frequency analysis of these cardiac signals using spatial filling index and Renyi's entropy has been proposed for detecting cardiac dysfunction. The ANOVA test was used to compare the different analyzing methods. The Renyi's entropy gives better result for the scalogram than the Wigner-Ville distribution. The evaluation of the proposed technique on a larger data set will improve the efficacy of the technique. It is left as future work to compare the different methods with more sophisticated statistical methods, such as post hoc comparisons. It is hoped that the graphical representation along with its corresponding analytical index and Renyi's entropy proposed here will find potential applications in computer analysis of cardiac patients' status in intensive care units.
